# *Turicibacter* fermentation enhances the inhibitory effects of *Antrodia camphorata* supplementation on tumorigenic serotonin and Wnt pathways and promotes ROS-mediated apoptosis of Caco-2 cells

**DOI:** 10.3389/fphar.2023.1203087

**Published:** 2023-08-16

**Authors:** Ting-Chun Lin, Anand Soorneedi, Yingxue Guan, Ying Tang, Eleanor Shi, Matthew D. Moore, Zhenhua Liu

**Affiliations:** ^1^ Department of Nutrition, School of Public Health and Health Sciences, University of Massachusetts, Amherst, MA, United States; ^2^ Department of Food Science, University of Massachusetts, Amherst, MA, United States; ^3^ Chobanian and Avedisian School of Medicine, Boston University, Boston, MA, United States; ^4^ UMass Cancer Center, University of Massachusetts Chan Medical School, Worcester, MA, United States

**Keywords:** mushroom polysaccharide, microbiome, *Turicibacter*, serotonin, Wnt/βcatenin signaling, reactive oxygen species, colorectal cancer

## Abstract

**Introduction:** Diet-induced obesity has been shown to decrease the abundance of *Turicibacter*, a genus known to play a role in the serotonin signaling system, which is associated with colorectal tumorigenesis, making the presence of *Turicibacter* potentially influential in the protection of intestinal tumorigenesis. Recently, *Antrodia camphorata* (AC), a medicinal fungus native to Taiwan, has emerged as a promising candidate for complementary and alternative cancer therapy. Small molecules and polysaccharides derived from AC have been reported to possess health-promoting effects, including anti-cancer properties.

**Methods:** Bacterial culture followed with cell culture were used in this study to determine the role of *Turicibacter* in colorectal tumorigenesis and to explore the anti-cancer mechanism of AC with *Turicibacter* fermentation.

**Results:** *Turicibacter* fermentation and the addition of AC polysaccharide led to a significant increase in the production of nutrients and metabolites, including α-ketoglutaric acid and lactic acid (*p* < 0.05). Treatment of *Turicibacter* fermented AC polysaccharide was more effective in inhibiting serotonin signaling-related genes, including *Tph1, Htr1d, Htr2a, Htr2b*, and *Htr2c* (*p* < 0.05), and *Wnt*-signaling related protein and downstream gene expressions, such as phospho-GSK-3β, active β-catenin, *c-Myc, Ccnd1, and Axin2* (*p* < 0.05). Additionally, it triggered the highest generation of reactive oxygen species (ROS), which activated PI3K/Akt and MAPK/Erk signaling and resulted in cleaved caspase-3 expression. In comparison, the treatment of AC polysaccharide without *Turicibacter* fermentation displayed a lesser effect.

**Discussion:** Our findings suggest that AC polysaccharide effectively suppresses the tumorigenic serotonin and *Wnt*-signaling pathways, and promotes ROS-mediated apoptosis in Caco-2 cells. These processes are further enhanced by *Turicibacter* fermentation.

## 1 Introduction

Colorectal cancer (CRC) is the third most commonly diagnosed cancer in both men and women and the second leading cause of cancer-related death worldwide (Siegel et al., 2022). In the United States, nearly 153,000 new cases are expected to be diagnosed in 2023, which translates to approximately one in 24 people developing this disease during their life. Additionally, there were approximately 52,000 CRC-related deaths per year, making it the second leading cause of cancer fatalities in the country (American Cancer Society, 2023).

The widely recognized risk factors of CRC include age, genetic and environmental factors (Thanikachalam and Khan, 2019). Among these identified risk factors, diet is one of the most controllable factors and has been studied extensively. In a meta-analysis, certain foods that possess pro-inflammatory potentials identified by the Dietary Inflammatory Index (DII), such as energy, total fat, trans fat, cholesterol, and saturated fatty acids, are associated with an increased risk of CRC. A 7% higher risk of CRC was reported for each 1-point increase in the DII score (Shivappa et al., 2014; Shivappa et al., 2017). It is reported that high-fat diets alter the intestinal microbial composition and short-chain fatty acid (SCFA) formation, which can promote intestinal tumorigenesis (Schulz et al., 2014; Chen et al., 2023). The Western-style diet, characterized by high saturated fat content, significantly promotes the obesity epidemic and CRC (Kopp, 2019; O'Neill et al., 2016; Benninghoff et al., 2020; Mehta et al., 2017). Therefore, understanding the mechanisms underlying diet-induced colorectal tumorigenesis can help practitioners develop strategies to reduce the increased risk of CRC associated with the obese population in Western countries, which has reached an epidemic level (Hruby and Hu, 2015).

A healthy gut microbiome, known as eubiosis, can protect the gastrointestinal tract from damage arising from daily digestion. In contrast, an imbalanced microbiome, known as dysbiosis, facilitates systemic and local inflammation and a microenvironment favoring colorectal tumorigenesis (Rebersek, 2021). Limited but fascinating data indicates that high-fat diets attenuate the abundance of genus *Turicibacter* in the gut microbiome (Everard et al., 2014; Guo et al., 2017), but little is known about the role of *Turicibacter* in CRC or any other gastrointestinal disease. Some studies suggested that the depletion of *Turicibacter* might be linked to colitis and may be correlated with increased *Tnf* and *NF-κB1* expression and decreased intestinal butyrate level (Jones-Hall et al., 2015; Zhong et al., 2015; Liu et al., 2016). As evidence has shown that butyrate is capable of relieving metabolic syndrome, maintaining the intestinal barrier, reducing inflammation, and inhibiting the growth of CRC cells. The presence of *Turicibacter*, therefore, might be associated with anti-inflammatory and anti-cancer properties (Canani et al., 2011). In addition, limited work indicates that *Turicibacter* may play a role in the serotonin-signaling system, which is intimately affiliated with intestinal inflammation and cancer development (Mawe and Hoffman, 2013; Fung et al., 2019; Kannen et al., 2020; Ala, 2022). Nevertheless, the casual relationship between *Turicibacter* and CRC remains largely undefined.

Chemotherapy is the primary strategy for treating CRC in addition to surgical therapy and is often accompanied by side effects. Accordingly, the development of complementary and alternative medicine is urgent, especially the use of natural products as potential anti-cancer remedies. *Antrodia camphorata* (AC) is a medicinal fungus rich in complementary polysaccharides and bioactive components for a Western-style diet. It has been reported to suppress proliferation, metastasis and epithelial-to-mesenchymal transition and induce apoptosis in human CRC cells particularly by suppressing the Wnt/β-catenin signaling pathway (Park et al., 2013; Hseu et al., 2017; Wang et al., 2017; Ding et al., 2021). However, the molecular mechanisms behind the effects remain largely unclear and necessitate further investigation. In the present study, we cultured Caco-2 cells with a conditioned medium of *Turicibacter* culture to determine the role of *Turicibacter* in colorectal tumorigenesis and to explore the anti-cancer mechanism of AC with *Turicibacter* fermentation. This innovative approach aims to investigate whether *Turicibacter* can enhance the effects of AC through biotransformation by microorganisms (Chávarri et al., 2021). By understanding the molecular interactions between AC and *Turicibacter*, we hope to shed light on the potential synergistic anti-cancer properties of this combination.

## 2 Materials and methods

### 2.1 Reagents and chemicals

AC supplementation powder (freeze-dried powder of concentrated supernatant collected from AC culture media, contains 44% polysaccharide) was obtained from New Bellus Enterprises Co., Ltd. (Tainan, Taiwan). Gibco™ Dulbecco’s modified Eagle medium (DMEM), Gibco™ fetal bovine serum (FBS), Gibco™ phosphate buffered saline, Gibco™ trypsin-EDTA, Gibco™ penicillin-streptomycin, TRIzol^®^ reagent, Applied Biosystems™ cDNA Reverse Transcription Kit, SYBR™ Green Master Mix, Pierce™ BCA Protein Assay Kit, Pierce™ Protease and Phosphatase Inhibitor, and Image-IT™ LIVE Green Reactive Oxygen Species Detection Kit were purchased from Thermo Fisher Scientific Co. (Waltham, MA). 3-(4,5-Dimethyl-2-thiazolyl)-2,5-diphenyl-2H-tetrazolium Bromide (MTT), puromycin dihydrochloride, RIPA lysis buffer, bovine serum albumin (BSA), and N-Acetyl-L-cysteine (NAC) were purchased from MilliporeSigma (Burlington, MA). Luciferase Assay System was purchased from Promega Co. (Madison, WI). Cignal Lenti TCF/LEF Reporter (luc), Cignal Lenti Negative Control (luc), and SureENTRY™ Transduction Reagent were purchased from Qiagen Inc. (Germantown, MD). Antibodies against β-catenin, p-NF-κB p65 (Ser536), p-Akt (Ser473), p-Mek (Ser221), p-Erk1/2 (Thr202/Tyr204), p-Gsk3β (Ser9), Cleaved caspase 3 (Asp175), GAPDH, and Anti-rabbit IgG, Horseradish peroxidase (HRP)-linked antibody were purchased from Cell Signaling Technology (Beverly, MA).

### 2.2 Bacterial strains and bacterial culture

A strain of *Turicibacter, Turicibacter sanguinis* DSM 14220 (originally isolated from human blood), was purchased from DSMZ (Braunschweig, Germany) and was used in this study. Frozen bacterial stocks were first retrieved in PYG TWEEN FA/GLC from Anaerobe systems (Morgan Hill, CA), then were grown and maintained in a non-commercial anaerobic bacterial culture medium with peptone, tryptone, yeast extract, casein hydrolysate, soluble starch, L-cysteine hydrochloride, bile salts, ferrous sulfate, hemin, salt solution, vitamin solution, Tween 80, and glucose with or without 1% AC supplementation at 37°C under anaerobic conditions. Supernatants were filter-sterilized and stored at −80°C until use. Metabolite profiling of supernatants was measured by hydrophilic interaction liquid chromatography (HILIC) positive and amide negative methods as previously described (Kimberly et al., 2017; Paynter et al., 2018).

### 2.3 Hydrophilic interaction liquid chromatography (HILIC)

AC supplementation powder is derived from the submerged fermentation of AC mycelium. Due to its complex composition, measuring all nutrients and bioactive constituents in the powder is challenging. To address this, HILIC method was employed to analyze identifiable components among four groups of cell-free supernatants from bacterial culture media: (1) bacterial culture medium only (CM), (2) medium with AC supplementation (ACCM), (3) *Turicibacter* cultured medium (TCM), and (4) *Turicibacter* cultured medium with AC supplementation (TACCM). This analysis can provide valuable information regarding the enrichment of nutrients from the AC supplementation and the differences in secondary metabolites after *Turicibacter* fermentation. Briefly, the supernatants of bacterial culture media were subjected to HILIC using a 2.1 × 100 mm, 3.5-μm XBridge™ amide column (Waters™, Milford, MA). Mobile phase A was 95:5 (v/v) water/acetonitrile, with 20 mM ammonium acetate and 20 mM ammonium hydroxide (pH 9.5). Mobile phase B was acetonitrile. For amide-negative mode, the chromatography system consisted of a 1260 Infinity autosampler (Agilent Technologies, Santa Clara, CA) connected to a 1290 Infinity HLPC binary pump system (Agilent Technologies, Santa Clara, CA). For the positive mode, the system utilized an HTS PAL autosampler (Leap Technologies, Morrisville, NC) connected to a 1260 HPLC binary system (Agilent Technologies, Santa Clara, CA). Metabolite quantification was determined by integrating peak areas using MassHunter QQQ Quant (Agilent Technologies, Santa Clara, CA) or MultiQuant™ software (Version 2.0, Sciex, Framingham, MA).

### 2.4 Cell line and cell culture

Human colon cancer cell line Caco-2 was obtained from American Tissue Culture Collection (ATCC, Manassas, VA). Caco-2 cells were maintained in DMEM supplemented with 10% FBS, 100 U/mL penicillin and 100 μg/mL streptomycin, and 1 mM sodium pyruvate at 37°C in a humidified incubator containing 5% CO_2_. After 48 h of culture, cells were sub-cultured at 80% confluence or were harvested for following cell experiments.

### 2.5 Cell viability assays

Caco-2 cells were seeded in 96-well plates with 2×10^4^ cells per well and incubated overnight. Subsequently, cells were starved in DMEM containing 0.5% FBS overnight. After starvation, cells were either mock-treated or pretreated with NAC (100 μM) for 1 h, then treated for 24 h with conditioned media of four cell-free supernatants collected from bacterial culture media: bacterial culture medium only (CM), medium with AC supplementation (ACCM), *Turicibacter* cultured medium (TCM), and *Turicibacter* cultured medium with AC supplementation (TACCM) or control medium. Conditioned media were prepared based on the percentage of supernatant (10%–90%) in the cell culture medium. Blanks were incubated with cell culture medium without seeding cells. Cell viability was determined by incubation with MTT (0.5 mg/mL) for 2 h. Formazan crystals were dissolved in DMSO, and the absorbance was measured by a BioTek Synergy H1 microplate reader (Agilent Technologies, Santa Clara, CA) at 570 nm wavelength. The average value obtained from the blanks was subtracted from the average values obtained from the treatment and control groups. Cell viability was expressed as a percentage compared to the control.

### 2.6 Quantitative real-time polymerase chain reaction (RT-PCR) analyses

Total RNAs from Caco-2 cells were extracted by using TRIzol^®^ reagent (Invitrogen™, Carlsbad, CA) according to the manufacturer’s instructions. Total RNA concentration as well as purity of RNA samples were determined by using NanoDrop™ Lite Spectrophotometer (Thermo Scientific™, Waltham, MA). The first-strand cDNAs were synthesized from RNA samples by using high-capacity cDNA reverse transcription kit (Applied Biosystems™, Carlsbad, CA). The relative expression of target genes was measured by using SYBR™ Green Master Mix (Applied Biosystems™, Carlsbad, CA) and ViiA™ 7 Real-Time PCR System (Applied Biosystems^®^, Carlsbad, CA) with the following conditions: hold stage starting at 50°C for 2 min, 95°C for 10 min, 40 cycles starting at 95°C for 15 s, 60°C for 1 min, melt curve stage starting at 95°C for 15 s, 60°C for 1 min, and 95°C for 15 s. The primers for target genes (*Tph1*, *Htr1d*, *Htr2a*, *Htr2b*, *Htr2c*, *c-Myc*, *Ccnd1*, *Axin2*, and *GAPDH*) were designed using PrimerBank (https://pga.mgh.harvard.edu/primerbank/). A list of the primers used can be found in Supplementary Table S1. The gene expression was normalized to the housekeeping gene *Gapdh* ( $\Delta Ct={Ct}_{-target\;gene}-{Ct}_{-Gapdh}$ ). Statistical analyses were performed based on ΔCt and relative expression is reported as 2^−ΔΔCT^, where $\Delta \Delta Ct={\Delta Ct}_{-experiment}-{\Delta Ct}_{-control}$ .

### 2.7 Immunoblotting

Western blot was performed to analyze protein expression as previously described (Lin et al., 2021). Briefly, 20 μg of protein from each group was loaded into 10% SDS-PAGE gel for protein separation, then was blotted onto Immun-Blot^®^ PVDF Membrane (Bio-Rad, Hercules, CA). After membranes were blocked in 5% BSA (Sigma-Aldrich, St. Louis, MO) in Tris Buffered Saline with Tween 20 (TBST) for 1 h, they were incubated overnight at 4°C with primary antibodies against β-catenin (1:1000), p-NF-κB p65 (1:1000), p-Akt (1:1000), p-Mek (1:1000), p-Erk1/2 (1:1000), p-Gsk3β (1:1000), cleaved caspase 3 (1:1000) and GAPDH (1:10000), followed by incubation with HRP-conjugated secondary antibody (1:5000) for 1 h at room temperature. Antibody binding was detected by incubating membranes with enhanced chemiluminescence substrate (Bio-Rad, Hercules, CA) and images were captured by Odyssey^®^ Fc Imaging System (LI-COR Biosciences, Lincoln, NE). Relative protein expression was quantified using Image J software after normalizing to the corresponding loading control. In general, phosphorylated levels of proteins reflect their functional impact. While total protein expression informs translational regulation, phosphorylation reflects changes at both translation and post-modification levels. Specifically, phosphorylated protein levels were measured using GAPDH as the loading control to gain insights into the total functional mechanisms underlying protein activity.

### 2.8 ROS generation assays

The presence of H_2_O_2_ reactive oxygen species in live cells was detected using the 5(6)-carboxy-2′,7′-dichlorodihydrofluorescein diacetate (carboxy-H_2_DCFDA) method. Generation of ROS was measured by using Image-IT™ LIVE Green Reactive Oxygen Species Detection Kit (Invitrogen™, Carlsbad, CA) according to the manufacturer’s instructions. Briefly, Caco-2 cells were seeded in a 96-well black plate with a clear bottom at a density of 2×10^4^ cells per well and were starved in DMEM containing 0.5% FBS. Then, the cells were either mock-treated or pretreated with NAC (100 μM) for 1 h. Subsequently, the cells were treated with conditioned media containing 30% supernatants collected from four groups of bacterial culture media or control medium for 6 h. After treatment, 25 μM carboxy-H_2_DCFDA was applied to cells and incubated for 30 min at 37°C. During the last 5 min of the incubation, 1.0 μM Hoechst 33342 was added to cells. Images were captured immediately after staining using the EVOS™ M5000 Imaging System (Invitrogen™, Carlsbad, CA), with wavelengths of 495 nm (excitation) and 529 nm (emission) for the detection of ROS generation, and wavelengths of 350 nm (excitation) and 461 nm (emission) for the detection of the cell nucleus. Relative fluorescence intensity was quantified using ImageJ software.

### 2.9 Lentiviral vector transduction

Caco-2 cells were transduced with the Wnt-signaling reporter gene using the Cignal Lenti TCF/LEF Reporter (luc) kit (Qiagen, Germantown, MD), while cells transduced with the Cignal Lenti Negative Control (luc) kit (Qiagen, Germantown, MD) served as the negative control. First, Caco-2 cells were grown in 35 × 10 mm cell culture dishes to 80% confluence, and then transduced with Cignal lentiviral particles diluted 1:10 in growth medium without antibiotics, along with SureENTRY™ Transduction Reagent (Qiagen, Germantown, MD). After 48 h, the viral particle-containing medium was replaced with growth medium containing 2 μg/mL puromycin, and cells were cultured for 2 weeks to select for a stably transduced cell line.

### 2.10 Firefly luciferase assays

Caco-2 cells transduced with the Cignal Lenti Reporter gene were seeded in 96-well plates at a density of 2×10^4^ cells per well and incubated overnight. The cells were then starved in DMEM containing 0.5% FBS overnight. After starvation, the cells were either mock-treated or pretreated with NAC (100 μM) for 1 h and then treated with conditioned media containing 30% supernatants collected from four groups of bacterial culture media or control medium for 6 h. After treatment, relative luciferase activity was measured using the Luciferase Assay System (Promega, Madison, WI) according to the manufacturer’s instructions, and the luminescence was detected using BioTek Synergy H1 microplate reader (Agilent Technologies, Santa Clara, CA).

### 2.11 Statistical analyses

Data are presented as means ± SEM. Statistical analyses were performed using SAS program (Version 9.4, SAS Institute, Cary, NC) and GraphPad Prism (Version 9, GraphPad Software, San Diego, CA). Two-tailed, unpaired *t*-test was applied to test the differences between two groups, one-way ANOVA was applied to test the differences among groups, and the dose-dependent relationship was assessed by the Cochran-Armitage test for trend.

## 3 Results

### 3.1 *Turicibacter* fermentation increased the production of bioactive substances

Table 1 shows the results of the HILIC analysis on supernatants, which demonstrated that the addition of AC supplementation to the bacterial culture medium significantly increased the levels of all the listed substances, particularly the levels of α-ketoglutaric acid (1.000 ± 0.099 vs. 10.014 ± 3.368), N-acetyl-L-glutamine (1.000 ± 0.111 vs. 4.090 ± 0.466), acetylcholine (1.000 ± 0.182 vs. 3.250 ± 0.086), N-acetyl-L-aspartic acid (1.000 ± 0.027 vs. 5.252 ± 0.036), oxalic acid (1.000 ± 0.150 vs. 94.349 ± 0.036), and butyric acid (1.000 ± 0.081 vs. 32.533 ± 1.171). Following *Turicibacter* fermentation, the levels of α-ketoglutaric acid (10.014 ± 3.368 vs. 25.650 ± 1.978), serotonin (1.927 ± 0.133 vs. 2.674 ± 0.165), and lactic acid (1.475 ± 0.032 vs. 41.809 ± 1.159) were further elevated. The full list of component analyses can be found in Supplementary Tables S2, S3.

**TABLE 1:** Bioactive Component analyses among 4 groups of supernatants by HILIC method. CM, fresh bacterial culture medium; TCM, *Turicibacter* culture medium; ACCM, fresh medium with AC; TACCM, *Turicibacter* culture medium with AC. Data was presented as relative mean AUC ±SEM compared to CM, *n* = 3. Red color indicates the statistical significances between CM and TCM or ACCM and TACCM. ^#^ *p* < 0.1, *p < 0.05 indicate the differences between CM and ACCM or TCM and TACCM.

	CM	TCM	ACCM	TACCM
α-Ketoglutaric acid	1.000 ± 0.099	2.031 ± 0.207	10.014 ± 3.368 *	25.650 ± 1.978*
N-Acetyl-L-Glutamine	1.000 ± 0.111	0.838 ± 0.056	4.090 ± 0.466 *	5.045 ± 0.397 *
N-Acetyl-L-Glutamic acid	1.000 ± 0.142	0.984 ± 0.164	1.692 ± 0.290^**#**^	2.470 ± 0.087 *
Dimethylglycine	1.000 ± 0.011	1.265 ± 0.064	1.817 ± 0.175 *	2.202 ± 0.025 *
Serotonin	1.000 ± 0.069	1.190 ± 0.080	1.927 ± 0.133 *	2.674 ± 0.165*
Choline	1.000 ± 0.005	1.226 ± 0.045	1.761 ± 0.133 *	2.023 ± 0.035 *
Acetylcholine	1.000 ± 0.182	1.039 ± 0.168	3.250 ± 0.086 *	3.533 ± 0.103 *
N-Acetyl-L-Aspartic acid	1.000 ± 0.027	1.556 ± 0.056	5.252 ± 0.036 *	5.602 ± 0.224 *
Citric acid/Isocitric acid	1.000 ± 0.078	1.029 ± 0.082	1.410 ± 0.029 *	1.479 ± 0.059 *
Oxalic acid	1.000 ± 0.150	1.616 ± 0.154	94.349 ± 0.036 *	114.887 ± 16.008 *
Lactic acid	1.000 ± 0.039	60.873 ± 0.623	1.475 ± 0.032 *	41.809 ± 1.159*
Butyric acid	1.000 ± 0.081	0.669 ± 0.192	32.533 ± 1.171 *	28.020 ± 2.694 *

### 3.2 *Turicibacter* metabolites of *Antrodia camphorata* supplementation promoted cytotoxic effect against Caco-2 cells

**FIGURE 1:**
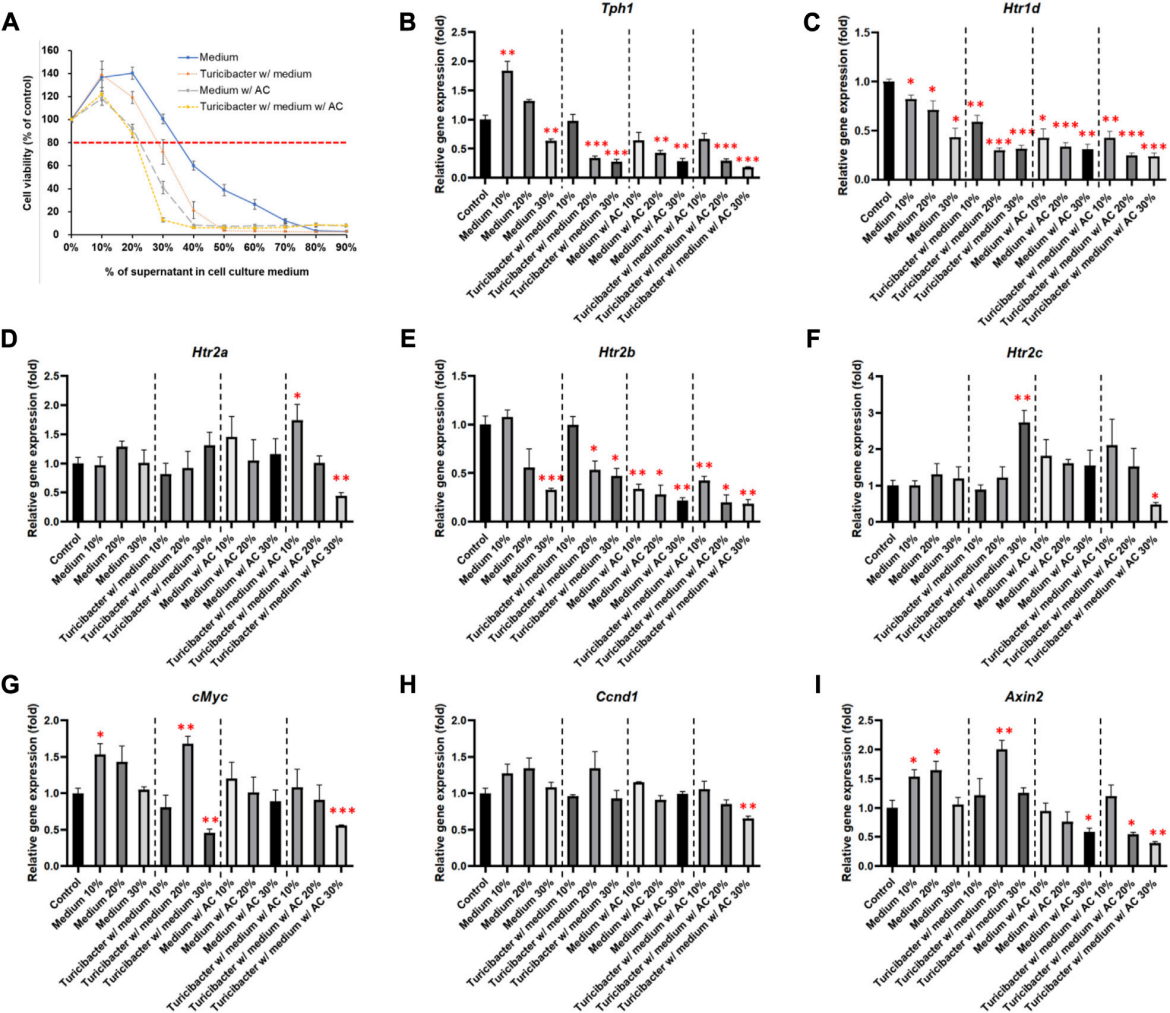
Cell viability and gene expression levels of Caco-2 cells cultured in conditioned medium. **(A)** Cell viability of Caco-2 cells cultured in conditioned medium with the supernatant of fresh bacterial culture medium, *Turicibacter* culture medium, fresh medium with AC, and *Turicibacter* culture medium with AC for 24 h **(B–I)** Gene expression levels of *Tph1*, *Htr1d*, *Htr2a*, *Htr2b*, *Htr2c*, *c-Myc*, *Ccnd1*, and *Axin2* in Caco-2 cells after 6 h of treatment by conditioned medium. Data was presented as mean ± SEM, *n* = 3. *p < 0.05, **p < 0.01, ***p < 0.001, as compared to the control. Abbreviation: w/, with; w/o, without.

To determine the appropriate treatment condition, we treated Caco-2 cells with conditioned media containing different percentages of supernatants collected from four groups of bacterial culture media: CM, TCM, ACCM, and TACCM. Our results showed that, in all four groups, cell viability started to decrease as the percentage of supernatant in the conditioned medium increased beyond 20% when compared to untreated cells. When the percentage of supernatants (CM, TCM, ACCM, and TACCM) fell between 10% and 30%, all these conditioned media only resulted in modest cytotoxic responses with <20% inhibition of cell viability (Figure 1A). Therefore, we decided to conduct further experiments by treating cells with conditioned media containing 10%–30% of supernatants.

Among the treatments of the four groups of conditioned media, supernatants from TACCM exhibited the most cytotoxicity, followed by supernatants from ACCM, TCM, and CM (Figure 1A).

### 3.3 *Turicibacter* metabolism enhanced the inhibitory effects of *Antrodia camphorata* supplementation on tumorigenic serotonin and Wnt pathways

As shown in Figure 1, treatments of the four groups of conditioned media containing 20% and 30% of supernatant dose-dependently suppressed *Tph1* (tryptophan hydroxylase 1) expression, which is the rate-limiting enzyme of serotonin synthesis (*p* < 0.01, Figure 1B). Moreover, the expression of *Htr1d* and *Htr2b* was also decreased dose-dependently (*p* < 0.05, Figures 1C, E), whereas the expression of *Htr2a* and *Htr2c* was significantly inhibited only by the treatment of conditioned media containing 30% of supernatant from TACCM (*p* < 0.05, Figures 1D, F).

Regarding the expression of Wnt-signaling downstream genes, *c-Myc* expression was significantly suppressed by treating with conditioned media containing 30% of supernatant from TCM and TACCM (*p* < 0.01, Figure 1G). *Ccnd1* expression was significantly suppressed only by treating with conditioned media containing 30% of supernatant from TACCM (*p* < 0.01, Figure 1H). *Axin2* expression was significantly suppressed by treating with conditioned media containing 30% of supernatant from ACCM and TACCM (*p* < 0.05, Figure 1I).

**FIGURE 2:**
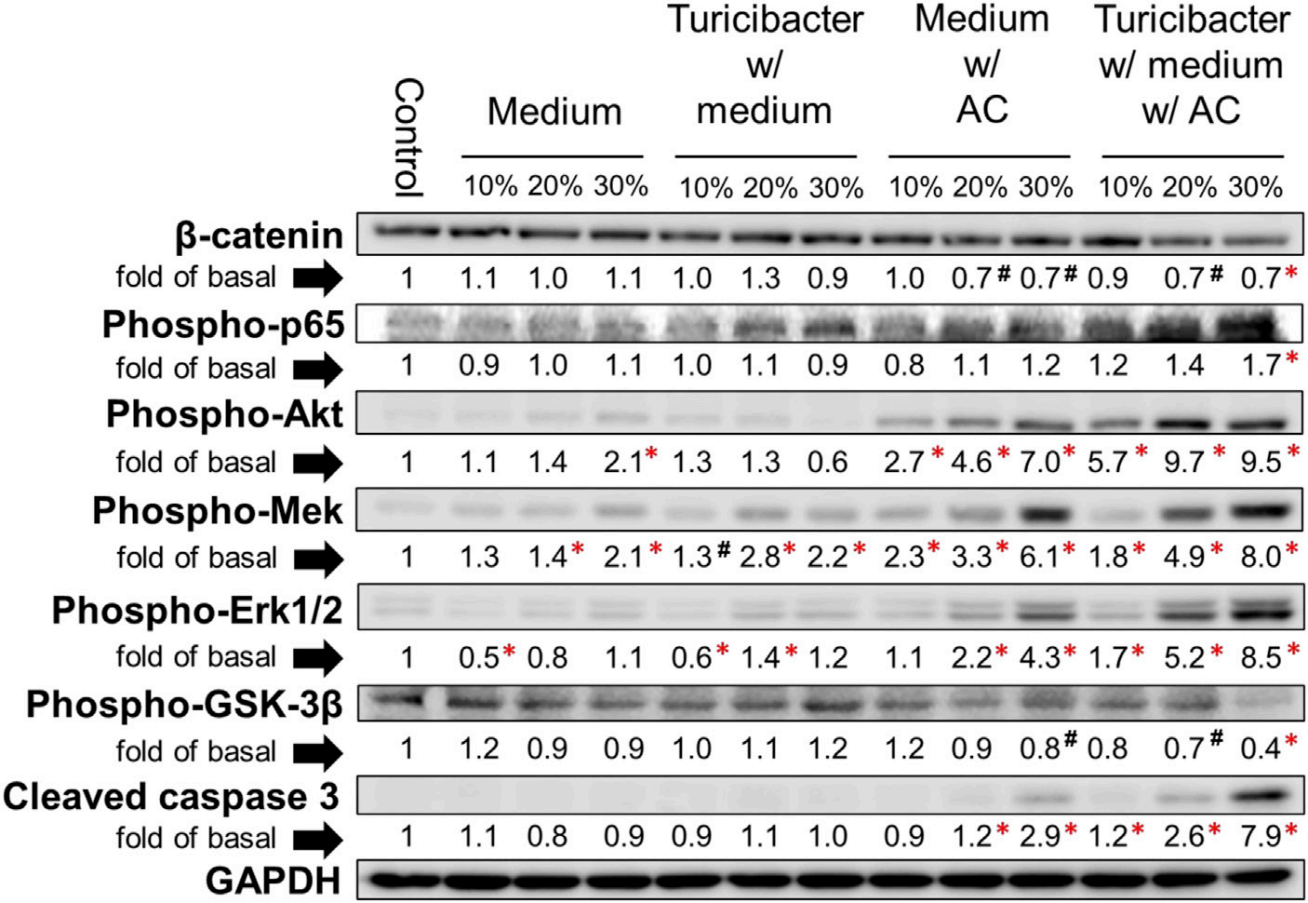
Protein expression of Caco-2 cells treated with conditioned medium. Western blots show the effects of conditioned medium treatment on the expression of β-catenin, phospho-p65, phospho-Akt, phospho-Mek, phospho-Erk1/2, phospho-Gsk-3β, and cleaved caspase 3 in Caco-2 cells. Data was presented as mean ± SEM, *n* = 3. ^#^ *p* < 0.1, *p < 0.05, as compared to the control. Abbreviation: w/, with; w/o, without.

The results of immunoblotting showed that expression of protein involved in Wnt-signaling, such as β-catenin and p-Gsk-3β, was significantly inhibited only by the treatment of conditioned media containing 30% of supernatant from TACCM (*p* < 0.05, Figure 2). In contrast, the protein expressions of p-Akt, p-Mek, and p-Erk1/2 were dose-dependently increased upon treatment with conditioned media containing 10%–30% of supernatant from ACCM and TACCM, with the TACCM groups showing a stronger promotion effect (*p* < 0.05, Figure 2).

### 3.4 *Turicibacter* metabolites of *Antrodia camphorata* supplementation promoted ROS generation and ROS-induced cell apoptosis

**FIGURE 3:**
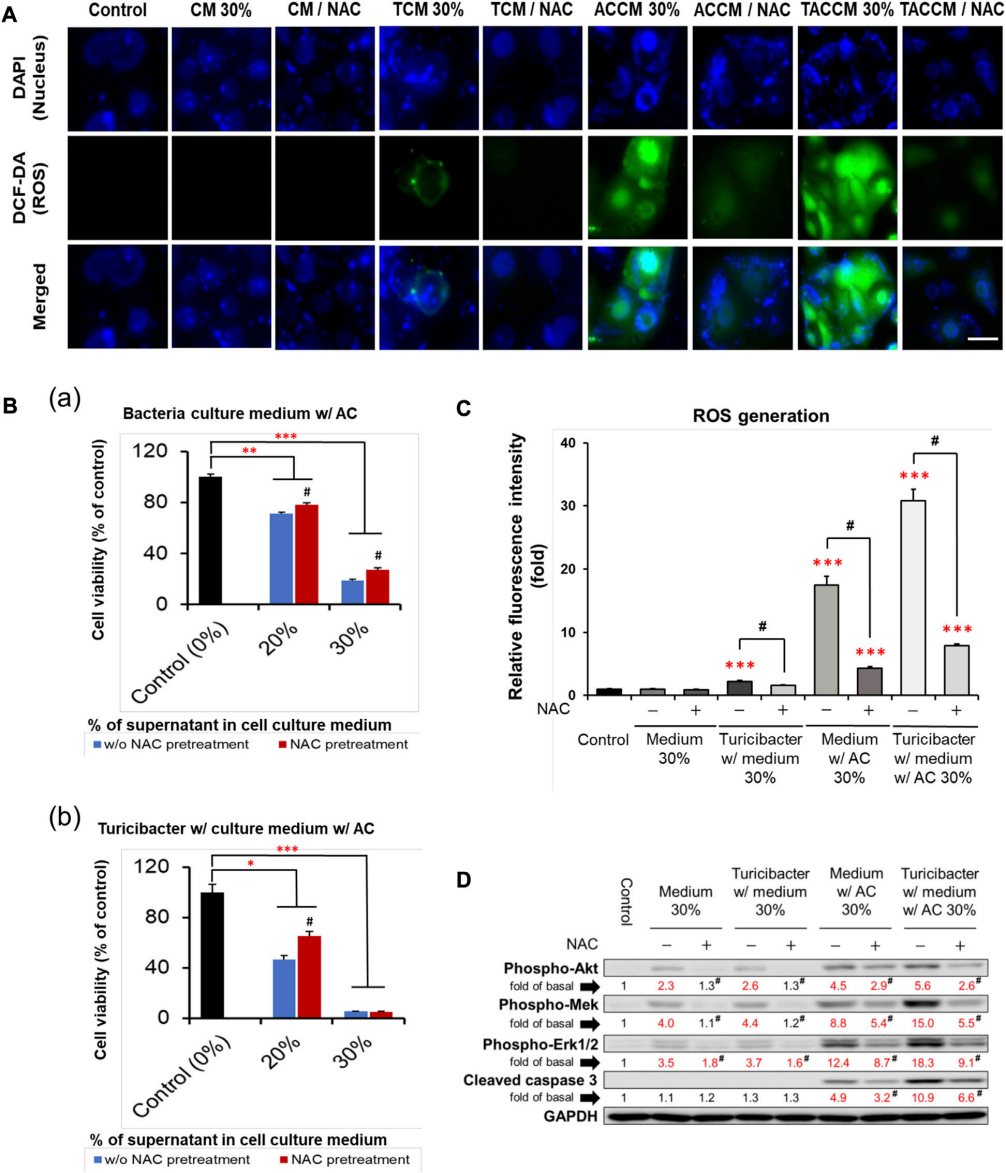
ROS generation and apoptosis of Caco-2 cells cultured in conditioned medium with and without pre-treatment of ROS inhibitor NAC. **(A)** Fluorescence microscopy of live Caco-2 cells untreated (control) or treated with conditioned medium with or without NAC (100 μM) pre-treatment. Carboxy-H_2_DCFDA was loaded to detect H_2_O_2_ in live cells (green color), and Hoechst 33342 was used for nucleic acid stain (blue color). **(B)** Cell viability of Caco-2 cells cultured in conditioned medium ACCM **(a)** and TACCM **(b)** for 24 h with or without NAC (100 μM) pre-treatment. **(C)** Relative H_2_O_2_ generation levels of Caco-2 cells untreated (control) or treated with conditioned media with or without NAC (100 μM) pre-treatment. **(D)** Western blots show the effects of NAC (100 μM) pre-treatment on the expression of proteins in conditioned media-treated Caco-2 cells. Red color specifies the statistical significance as compared to the control. ROS, Reactive Oxygen Species; NAC, ROS inhibitor N-acetyl-l-cysteine. CM, fresh bacterial culture medium; TCM, *Turicibacter* culture medium; ACCM, fresh medium with AC; TACCM, *Turicibacter* culture medium with AC. Data was presented as mean ± SEM, *n* = 3. *p < 0.05, **p < 0.01, ***p < 0.001, as compared to the control; ^#^ *p* < 0.05 indicates the impact of NAC. Abbreviation: w/, with; w/o, without.

As illustrated in Figures 3A, C, compared to the untreated control group, treatment with conditioned media containing 30% of supernatant from TCM, ACCM, and TACCM stimulated ROS generation. The TACCM group showed the greatest promoting effect, followed by ACCM and TCM groups (*p* < 0.001). Pretreatment with NAC, a ROS production inhibitor, effectively attenuated the ROS production induced by the treatments (*p* < 0.05).

The results of cell viability assays showed that NAC pretreatment partially abolished the cytotoxicity effect of conditioned media containing supernatant from ACCM and TACCM, with the strongest protective effect observed in the treatment of conditioned medium containing 20% of supernatant from TACCM (*p* < 0.05, Figure 3B). The increased expression of cleaved caspase 3 promoted by the treatment of conditioned media containing supernatant from ACCM and TACCM was significantly attenuated by pre-treatment of the ROS inhibitor NAC (*p* < 0.05, Figure 3D). This result reiterated the dose-dependent elevation of cleaved caspase 3 upon treatment with conditioned media containing 10%–30% of supernatant from ACCM and TACCM, with the TACCM groups showing a stronger promotion effect (*p* < 0.05, Figure 2).

### 3.5 Conditioned medium of *Turicibacter* cultured with *Antrodia camphorata* supplementation inhibited tumorigenic serotonin and Wnt pathways independent of ROS production

**FIGURE 4:**
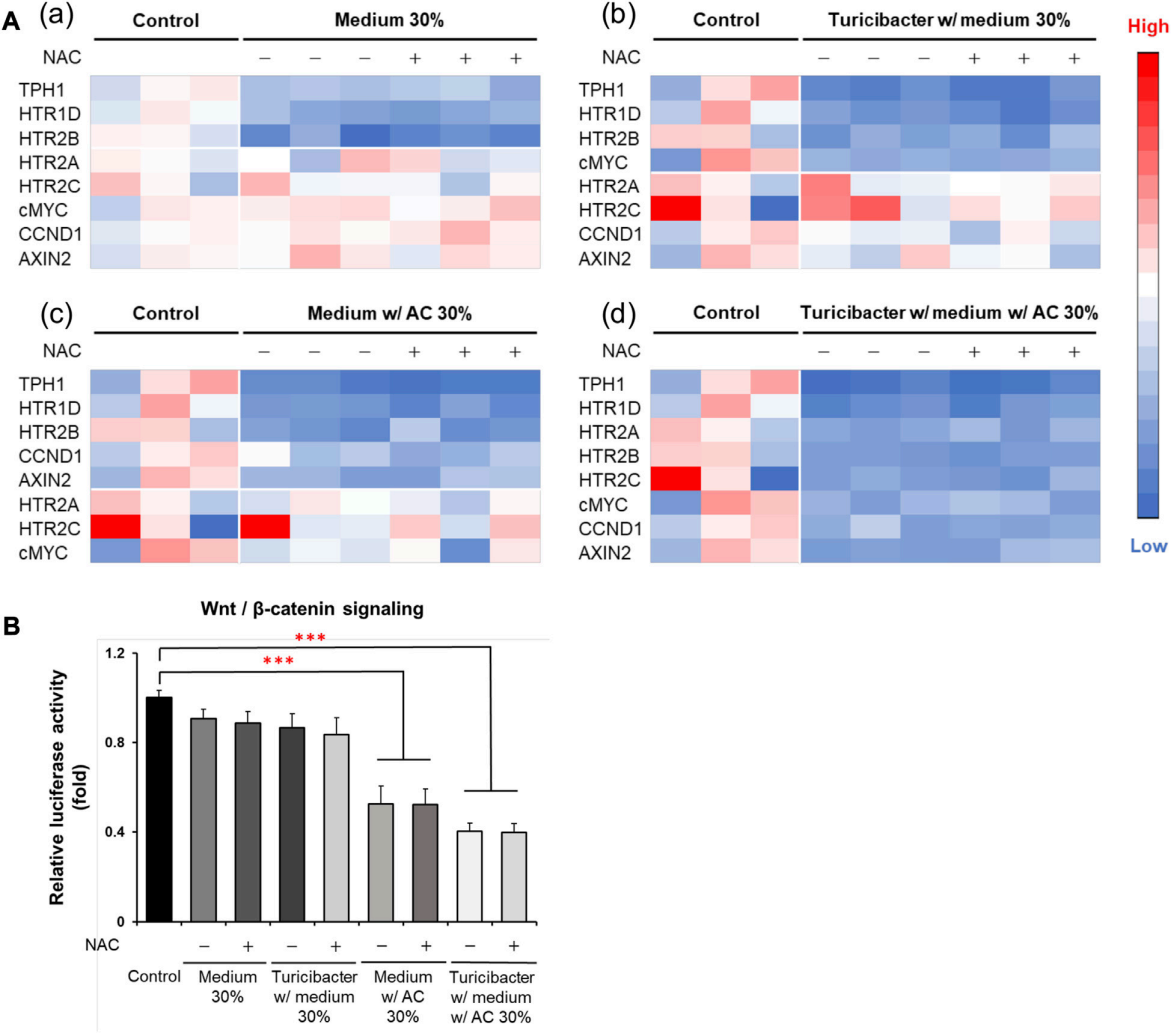
Heatmaps for expression of genes related to serotonin and Wnt pathways and Wnt/β-catenin signaling in Caco-2 cells cultured in conditioned media with and without ROS inhibitor NAC pre-treatment. **(A)** Gene expression heatmaps for the effect of NAC (100 μM) pre-treatment on expression of genes related to serotonin and Wnt pathways in conditioned medium-treated Caco-2 cells. **(B)** Wnt/β-catenin signaling, as indicated by TCF/LEF luciferase reporter gene activation, in response to NAC (100 μM) pre-treatment in conditioned medium-treated Caco-2 cells. ROS, Reactive Oxygen Species; NAC, ROS inhibitor N-acetyl-l-cysteine. Data was presented as mean ± SEM, *n* = 3. ***p < 0.001, as compared to the control. Abbreviation: w/, with; w/o, without.

Although ROS inhibitor NAC inhibited ROS production and attenuated ROS-mediated apoptosis, which was otherwise promoted by the conditioned medium from *Turicibacter* fermentation of *Antrodia camphorata* supplementation (Figure 3), NAC pretreatment (100 μM) did not affect the activation of tumorigenic serotonin and Wnt-signaling pathways when exposed to conditioned media (Figure 4). Caco-2 cells treated with the supernatants from CM, TCM, ACCM, and TACCM inhibited the expression of genes related to the serotonin and Wnt pathways with the strongest suppression for TACCM, whereas the NAC pretreatment did not mediate this impact (Figure 4A). Similarly, the supernatants from CM, TCM, ACCM, and TACCM inhibited Wnt/β-catenin signaling, as indicated by TCF/LEF luciferase reporter gene activation, whereas the impact was independent to NAC pre-treatment (Figure 4B).

## 4 Discussion

Studies have shown that CRC patients have a distinct microbiome composition compared to healthy individuals, with alterations in the abundance of specific bacterial species (Vogtmann et al., 2016; Thomas et al., 2019). Accumulating evidence suggests that the composition and activity of the gut microbiome can affect the development and progression of CRC (Nyangale et al., 2012; Schwabe and Jobin, 2013; Irrazábal et al., 2014; Louis et al., 2014; Liu et al., 2021), whereas the mechanism(s) by which the microbiome affects CRC remains insufficiently apprehended. Emerging evidence suggests that microbiome modulation through dietary interventions, pre- and probiotics may be a potential preventive or therapeutic strategy for CRC (Cheng et al., 2020). The present study suggests the role of an understudied genus, *Turicibacter*, and the potential of a specific mushroom *Antrodia camphorata* in the inhibition of the tumorigenic serotonin and Wnt pathways and the promotion of ROS-mediated apoptosis in the Caco-2 CRC cell line.

Previously, we discovered that the abundance of the rarely explored *Turicibacter* was significantly reduced in the gut microbiome of high-fat diet-induced obese C57BL/6 mice and *APC* ^*+/1638N*^ mice, accompanied by elevated inflammation and Wnt-signaling activation (Liu et al., 2016; Guo et al., 2017). In the present study, based on the metabolite profiling of the medium supernatants by HILIC, it is not surprising that the addition of AC supplementation significantly enriched a great number of bioactive components (Table 1; Supplementary Tables S2, S3) in the cell-free supernatants of bacterial culture media (ACCM vs. CM and TACCM vs. TCM). It is also important to note that *Turicibacter* fermentation resulted in an increased number of certain metabolites including α-ketoglutaric acid and lactic acid regardless of AC supplementation (TCM vs. CM and TACCM vs. ACCM) and serotonin with the presence of AC supplementation (TACCM vs. ACCM) (Table 1).

α-ketoglutaric acid is a critical intermediate in the tricarboxylic acid (TCA) cycle, which is one of the most important pathways for energy metabolism. It was reported that the depletion of α-ketoglutaric acid and glutamine could activate Wnt-signaling and promote cancer dedifferentiation in CRC with *Apc* mutation in mouse and human *ex vivo* organoids, whereas the supplementation of α-ketoglutaric acid antagonized tumorigenesis through epigenetic reprogramming (Tran et al., 2020). α-ketoglutaric acid has also been shown in various cancer cell lines to induce ROS generation, leading to pyroptosis (Zhang et al., 2021). Moreover, α-ketoglutaric acid was shown to alleviate intestinal inflammation in C57BL/6 mice with chemically-induced CRC and was capable of increasing the abundance of anti-inflammatory and SCFA-producing bacteria (Li et al., 2019). In alignment with the previous findings, our results indicated that treatment of supernatants containing higher levels of α-ketoglutaric acid attenuated Wnt-signaling, increased ROS generation, and ROS-mediated apoptosis (Figures 2–4), implying that the role of *Turicibacter* mediates tumorigenic pathways via the intermediate metabolite α-ketoglutaric acid.

A previous report showed that *Turicibacter* could stimulate the production of serotonin in the gut of healthy individuals (Yano et al., 2015), and production was likely through short-chain fatty acids (Reigstad et al., 2015). Reciprocally, serotonin has also been reported to promote *Turicibacter* colonization (Fung et al., 2019). In this study, we did not observe that *Turicibacter* fermentation increased butyric acid production and only slightly increased serotonin in the medium; this is probably due to differences between the *in vitro* and *in vivo* models used. In contrast, this study clearly demonstrated that the conditioned medium of *Turicibacter* significantly suppressed the expression of *Tph1* and serotonin receptors in Caco-2 cells, with the addition of AC supplementation further enhancing this effect (Figures 1B–F). This suggests that *Turicibacter* and AC supplementation could potentially impact the development of CRC by altering serotonin signaling (Mawe and Hoffman, 2013; Ala, 2022). In fact, the serotonin pathway in CRC development is extremely complicated. Growing evidence suggests that serotonin plays a dual role in CRC development, with its presence promoting DNA damage repair in early stages but contributing to proliferation, angiogenesis, and metastasis in the later stages via receptor signaling or re-uptake transporters (Kannen et al., 2020). Notably, the activation of serotonin receptor (5-HT) 1 and 2 has been linked to the amplification of tumorigenic signaling pathways, such as β-catenin, MAPK, and AKT signaling (Karmakar and Lal, 2021; Ala, 2022). Previous research has also shown that treatment with a 5-HT_2A_ receptor antagonist, Pizotifen, inhibits proliferation and migration of HCT-116 cells through inhibiting the Wnt/β-catenin signaling pathway (Piao and Shang, 2019). Therefore, our results demonstrate that *Turicibacter* and AC supplementation suppress serotonin signaling, suggesting a potential anti-tumorigenic effect in Caco-2 cells.

Our results showed that treating Caco-2 cells with conditioned medium ACCM and TACCM dramatically increased ROS generation and ROS-mediated apoptosis as indicated by the increased expression of cleaved caspase 3, with the strongest effect observed in the latter treatment (Figure 3). In addition, we observed increased expression of MAPK/Erk and PI3K/Akt signaling by the conditioned media ACCM and TACCM (Figures 2, 3D). Though the activation of MAPK/Erk and PI3K/Akt signals typically promote cell proliferation, survival, and tumorigenesis, emerging evidence suggests that the sustained accumulation of Erk1/2 above a certain threshold in the cytoplasm and nucleus, triggered by stimuli such as ROS, can lead to cell apoptosis (Sugiura et al., 2021). In addition, it has been suggested that there is a positive feedback loop between ROS and abnormally activated PI3K/Akt signaling, whereby hyperactive PI3K/Akt signaling may contribute to the production of excessive ROS and ROS-mediated apoptosis (Koundouros and Poulogiannis, 2018), which is consistent with what is observed in this study. In line with our results, previous research has shown that piperlongumine, a bioactive compound derived from long peppers, induces ROS-mediated apoptosis through the MAPK/Erk pathway in HT-29 cells (Randhawa et al., 2013).

Though previous research has shown that CRC cells exhibit higher ROS levels compared to normal colon cells, which contribute to cancer cell growth, the excessive production of ROS over a threshold can also lead to cancer cell apoptosis (Zeng et al., 2021). The elevated ROS levels in CRC cells are a result of dysregulated redox homeostasis. While normal cells maintain a balance to neutralize ROS damage, cancer cells experience disrupted redox balance, leading to moderate ROS levels that may support cancer cell survival (Liu et al., 2017). Notably, CRC cells display reduced activity of enzymes involved in antioxidant functions compared to normal colon cells (De Giani et al., 2022). This diminished antioxidant capacity in cancer cells makes them more susceptible to damage caused by excessive ROS or oxidative stress compared to normal cells. Therefore, targeting ROS-mediated apoptosis has emerged as a promising anti-cancer strategy due to this vulnerability in CRC cells. Inducing apoptosis through ROS enables selective targeting of cancer cells while sparing normal cells from adverse effects.

The vast majority (>90%) of CRC in humans possess over-activation of the Wnt pathway and there is strong evidence that this activation plays a pivotal role in colorectal carcinogenesis (Taketo, 2004; Klaus and Birchmeier, 2008; Network, 2012). The present study explicitly demonstrated that the *Turicibacter* fermentation together with AC supplementation (TACCM) diminished the Wnt/β-catenin signaling in Caco-2 cells, indicating an anti-colorectal tumorigenic function (Figures 1G–I and Figure 2). Moreover, this impact on Wnt/β-catenin signaling (serotonin pathway as well) is independent of ROS production and ROS-mediated apoptosis (Figures 3D, Figure 4).

In summary, based on our findings of the impacts on the serotonin pathway, Wnt/β-catenin signaling, and ROS-mediated apoptosis, AC supplementation exhibits an anti-CRC property which is further enhanced by *Turicibacter* fermentation. As such, this unique AC mushroom and the rarely explored *Turicibacter* have the potential to be developed into a complementary strategy for the prevention and treatment of CRC. Furthermore, previous safety assessments indicate that AC is safe for oral consumption, making it suitable for further investigation in animal experiments using a mouse model (Lin et al., 2015; EFSA Panel on Nutrition et al., 2022). Conducting an *in vivo* study will provide valuable insights into the potential therapeutic benefits of AC and *Turicibacter* fermentation on CRC, helping researchers understand their effects on tumor growth, metastasis, and other relevant parameters, and supporting the development of potential treatments for CRC.

## Data Availability

The original contributions presented in the study are included in the article/Supplementary Material, further inquiries can be directed to the corresponding author.
