# Supplementary material for: Turicibacter fermentation enhances the inhibitory effects of Antrodia camphorata supplementation on tumorigenic serotonin and Wnt pathways and promotes ROS-mediated apoptosis of Caco-2 cells
Source: Front Pharmacol. 2023 Aug 16;14:1203087. doi: 10.3389/fphar.2023.1203087 (PMC10469317; doi:10.3389/fphar.2023.1203087)
Supplement: Supplementary file 1 [file Table1.DOCX]

***Supplementary Material***

***Turicibacter* fermentation enhances the inhibitory effects of *Antrodia camphorata* supplementation on tumorigenic serotonin and Wnt pathways and promotes ROS-mediated apoptosis of Caco-2 cells**

**Ting-Chun Lin, Anand Soorneedi, Yingxue Guan, Ying Tang, Eleanor Shi, Matthew D. Moore, Zhenhua Liu^*^**

***Correspondence:**

Corresponding Author: Zhenhua Liu, Ph.D.

Email: zliu@nutrition.umass.edu; Tel: 1-413-545-1075; Fax: 1-413-545-1074

**Supplementary Tables**

| **Pathway** | **Gene** | **Forward Primer** | **Reverse Primer** |
| --- | --- | --- | --- |
| Serotonin signaling | *Tph1* | ACGTCGAAAGTATTTTGCGGA | ACGGTTCCCCAGGTCTTAATC |
|  | *Htr1d* | CTCCAACAGATCCCTGAATGC | CCTGGTGAGTAAGATGGTGGT |
|  | *Htr2a* | CTTTGTGCAGTCTGGATTTACCT | ACTGATATGGTCCAAACAGCAAT |
|  | *Htr2b* | TGATTTGCTGGTTGGATTGTTTG | ATGGATGCGGTTGAAAAGAGAA |
|  | *Htr2c* | CTAATTGGCCTATTGGTTTGGCA | CCACCATCGGAGGTATTGAAAA |
| Wnt signaling | *c-Myc* | GGCTCCTGGCAAAAGGTCA | CTGCGTAGTTGTGCTGATGT |
|  | *Ccnd1* | GCTGCGAAGTGGAAACCATC | CCTCCTTCTGCACACATTTGAA |
|  | *Axin2* | CAACACCAGGCGGAACGAA | GCCCAATAAGGAGTGTAAGGACT |
| Reference | *GAPDH* | GGAGCGAGATCCCTCCAAAAT | GGCTGTTGTCATACTTCTCATGG |

**Supplementary Table S1**. Primers for serotonin signaling and Wnt signaling related genes


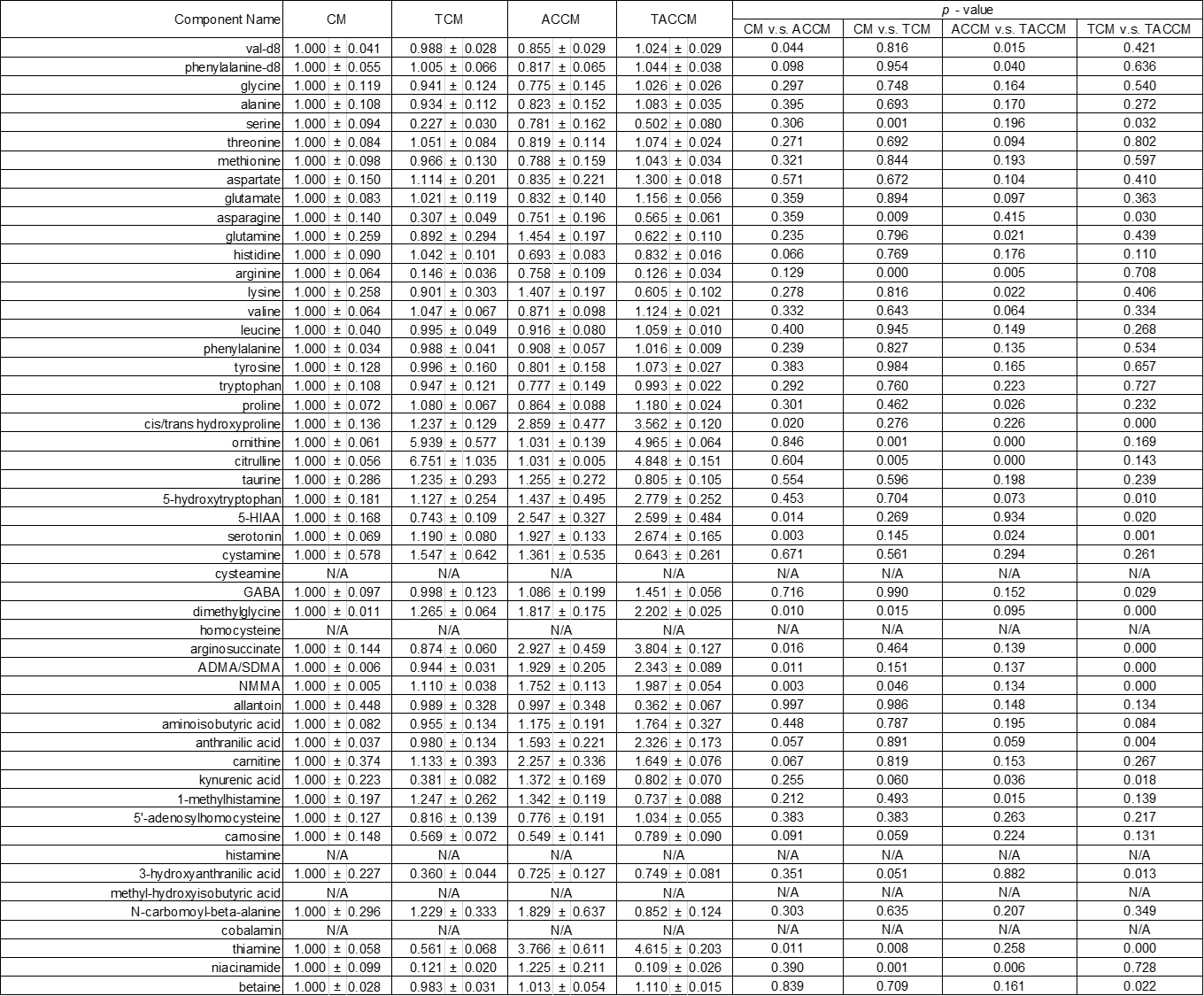


**Supplementary Table S2**. Bioactive component analyses among 4 groups of supernatants by HILIC method in positive mode. CM: fresh bacterial culture medium; TCM: *Turicibacter* culture medium; ACCM: fresh medium with AC; TACCM: *Turicibacter* culture medium with AC. Data was presented as relative mean AUC ± SEM compared to fresh bacteria culture medium, n=3.


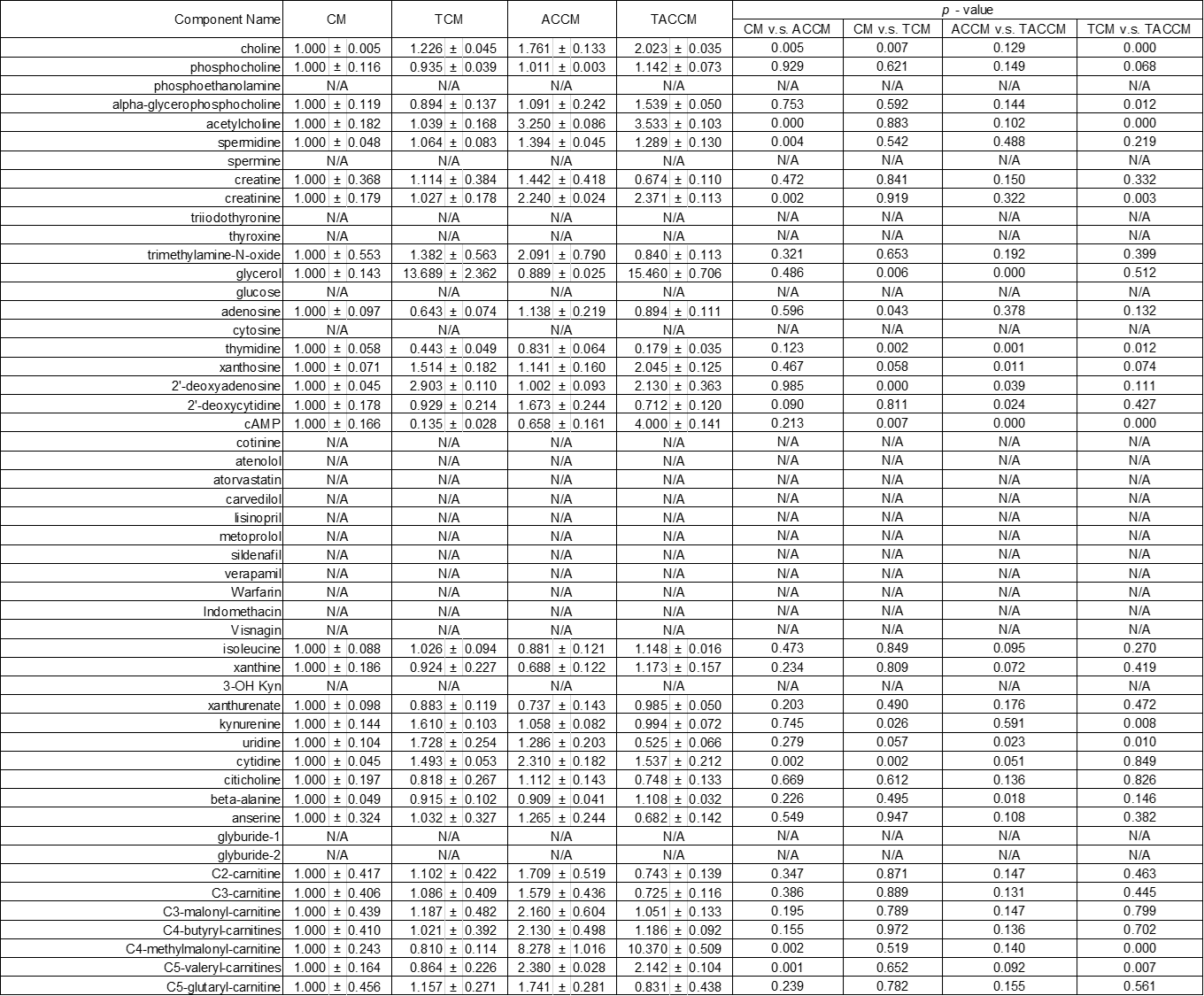


**Supplementary Table S2 (continued)**. Bioactive component analyses among 4 groups of supernatants by HILIC method in positive mode. CM: fresh bacterial culture medium; TCM: *Turicibacter* culture medium; ACCM: fresh medium with AC; TACCM: *Turicibacter* culture medium with AC. Data was presented as relative mean AUC ± SEM compared to fresh bacteria culture medium, n=3.


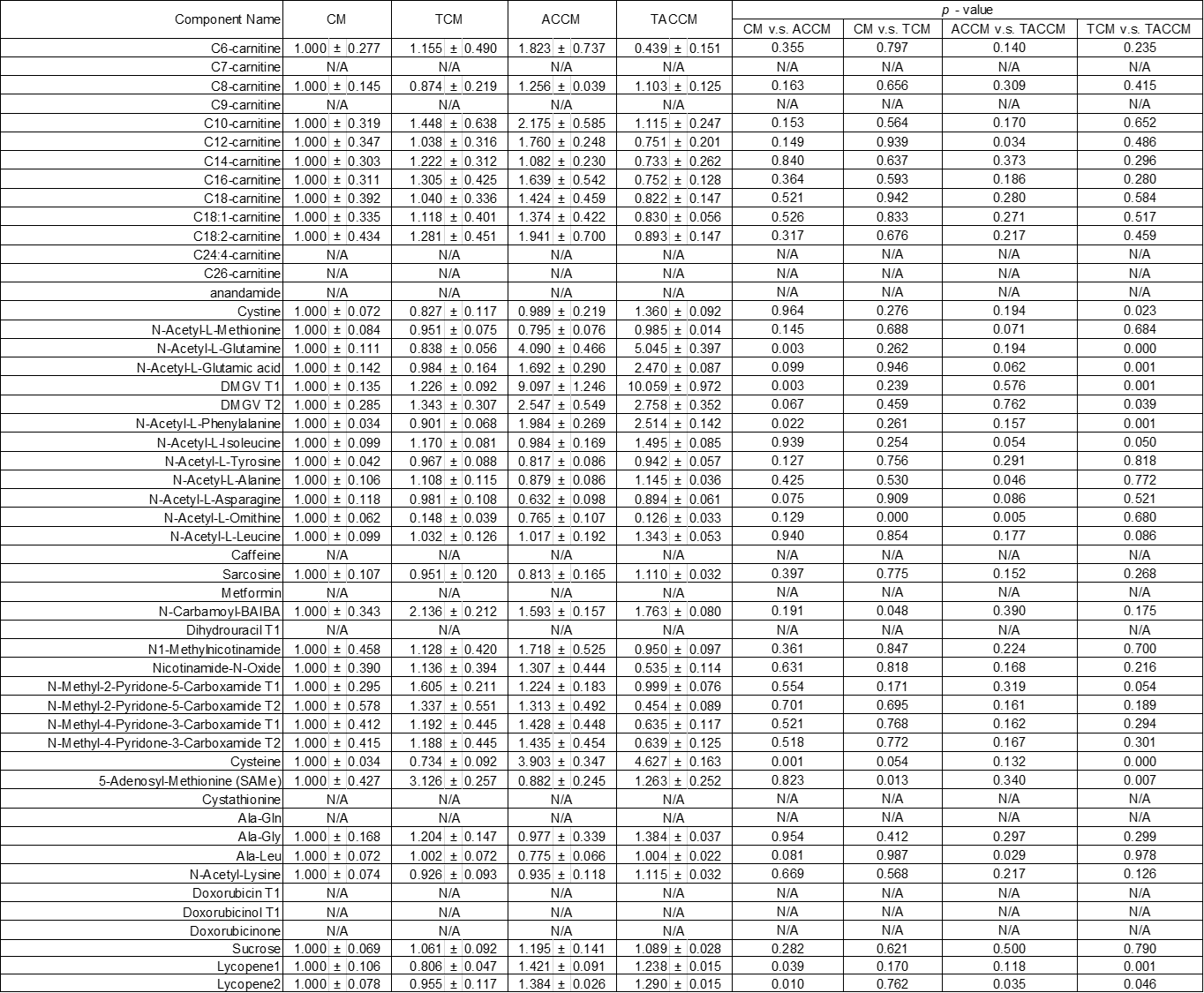


**Supplementary Table S2 (continued)**. Bioactive component analyses among 4 groups of supernatants by HILIC method in positive mode. CM: fresh bacterial culture medium; TCM: *Turicibacter* culture medium; ACCM: fresh medium with AC; TACCM: *Turicibacter* culture medium with AC. Data was presented as relative mean AUC ± SEM compared to fresh bacteria culture medium, n=3.


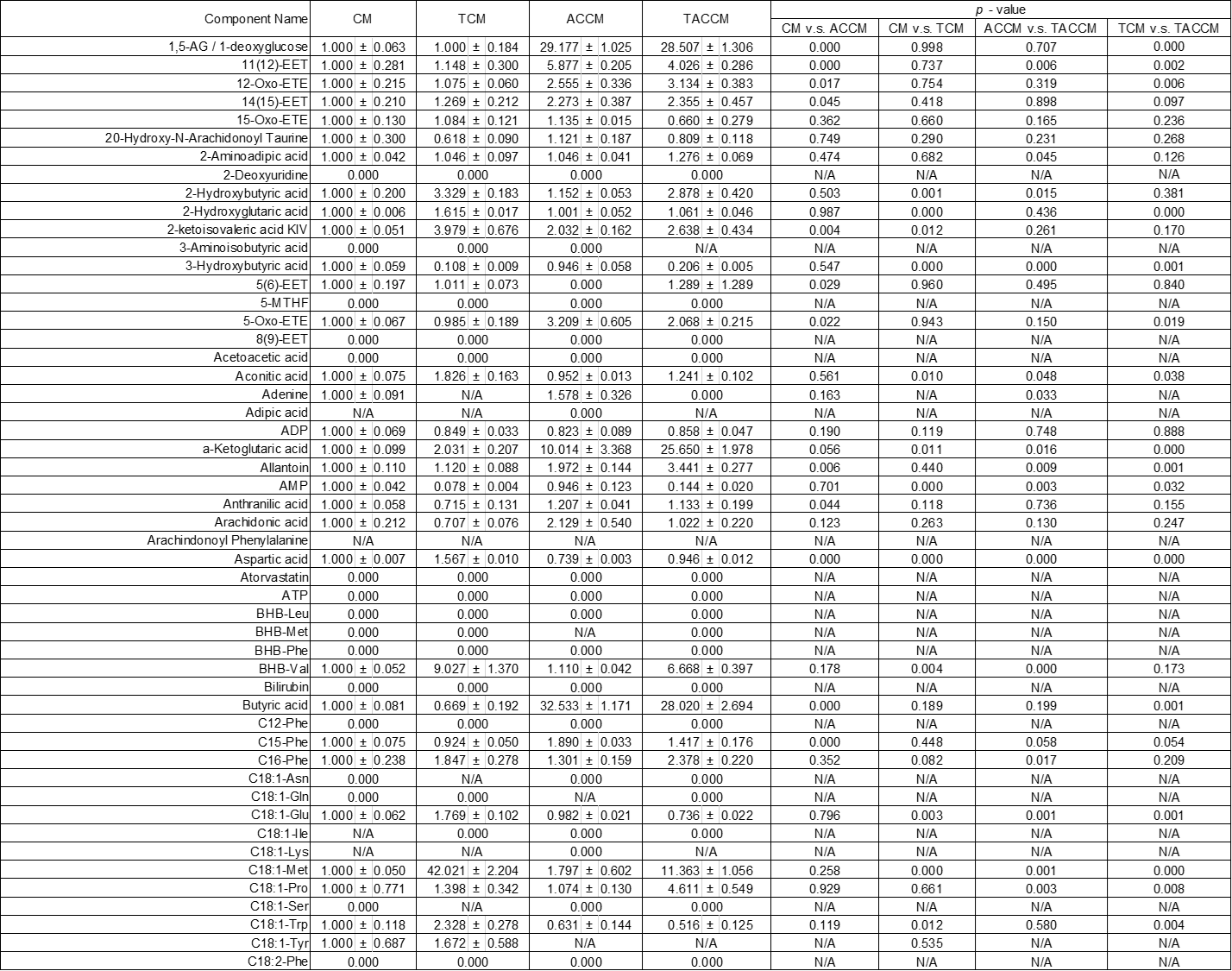


**Supplementary Table S3**. Bioactive component analyses among 4 groups of supernatants by HILIC method in negative mode. CM: fresh bacterial culture medium; TCM: *Turicibacter* culture medium; ACCM: fresh medium with AC; TACCM: *Turicibacter* culture medium with AC. Data was presented as relative mean AUC ± SEM compared to fresh bacteria culture medium, n=3.


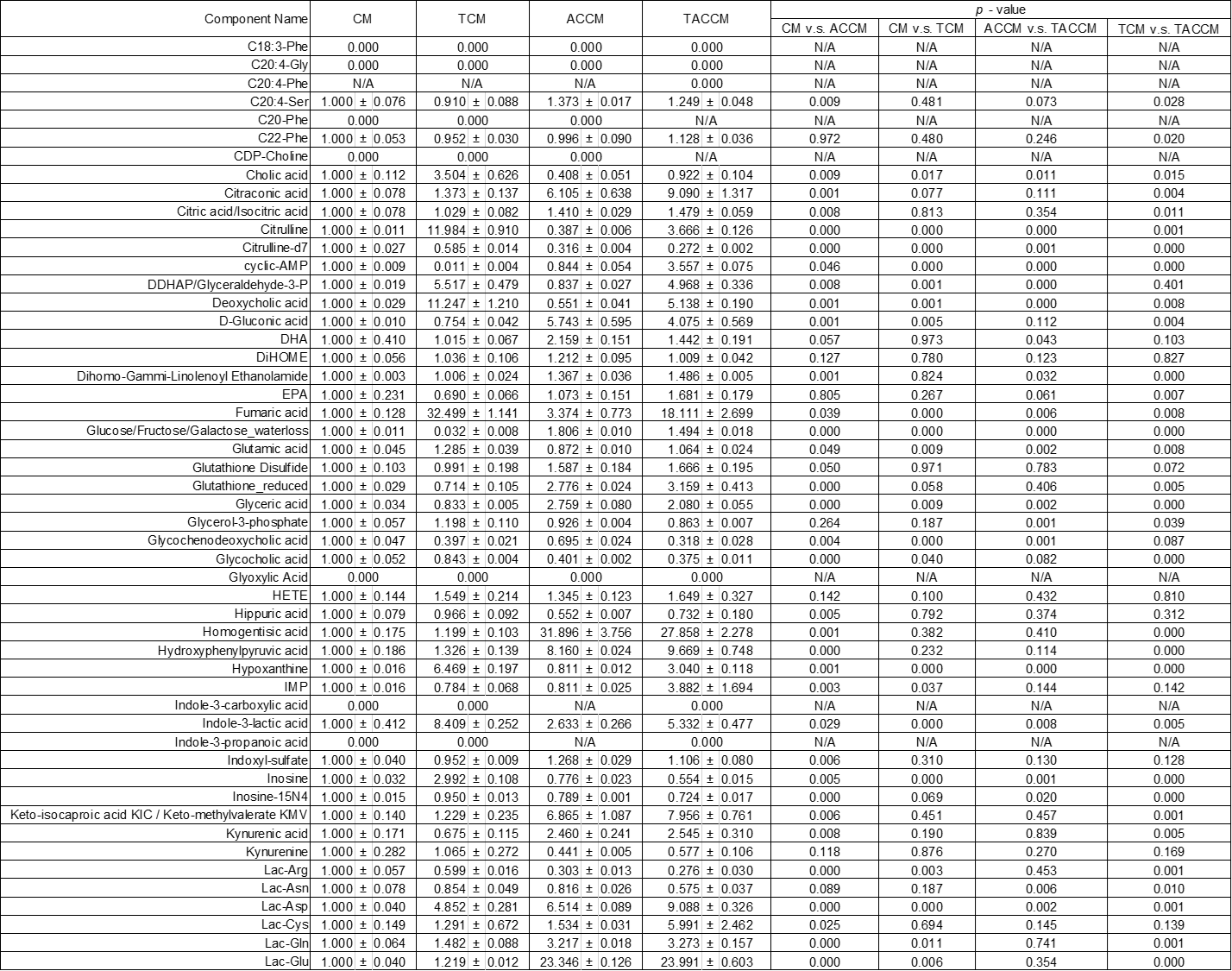


**Supplementary Table S3 (continued)**. Bioactive component analyses among 4 groups of supernatants by HILIC method in negative mode. CM: fresh bacterial culture medium; TCM: *Turicibacter* culture medium; ACCM: fresh medium with AC; TACCM: *Turicibacter* culture medium with AC. Data was presented as relative mean AUC ± SEM compared to fresh bacteria culture medium, n=3.


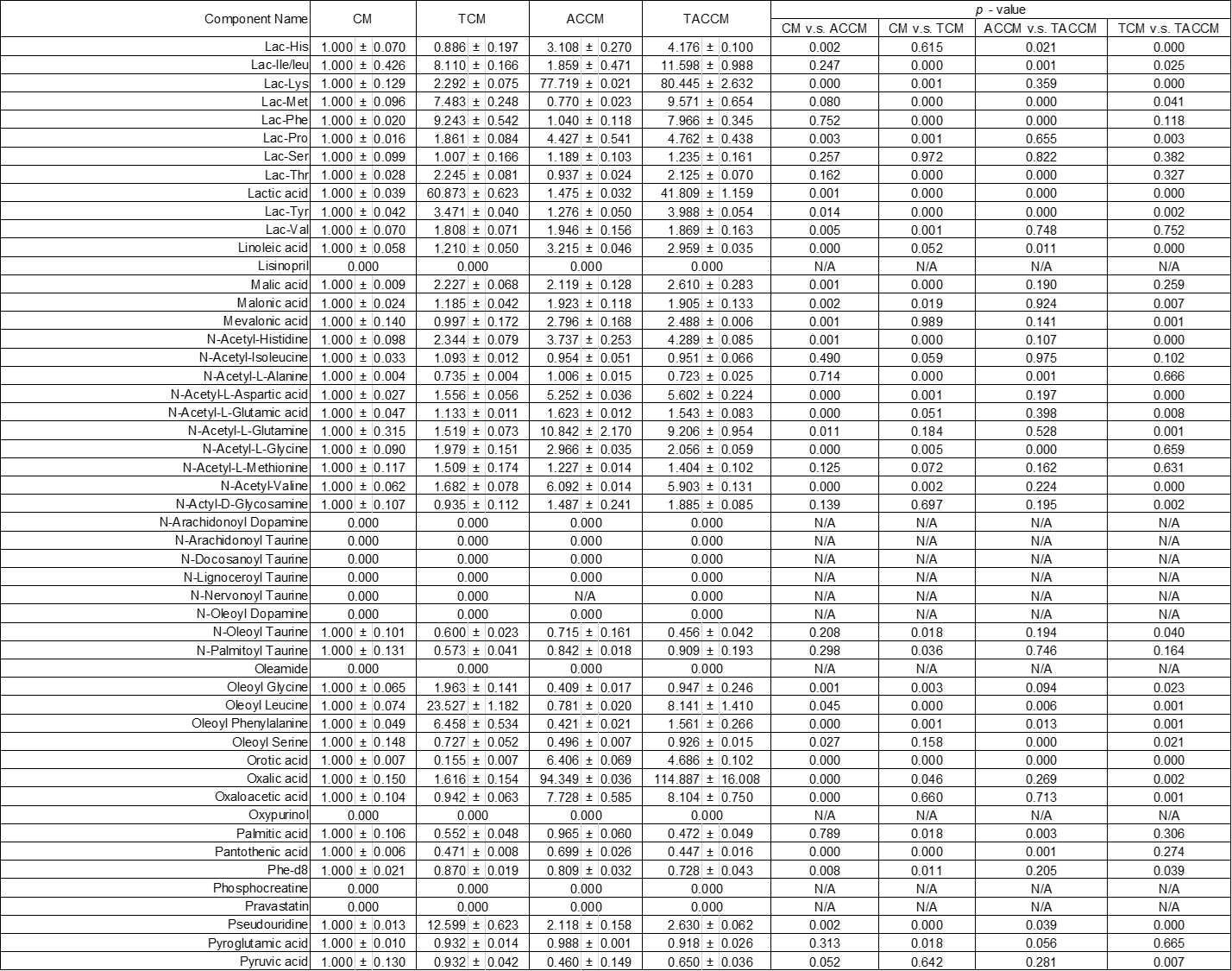


**Supplementary Table S3 (continued)**. Bioactive component analyses among 4 groups of supernatants by HILIC method in negative mode. CM: fresh bacterial culture medium; TCM: *Turicibacter* culture medium; ACCM: fresh medium with AC; TACCM: *Turicibacter* culture medium with AC. Data was presented as relative mean AUC ± SEM compared to fresh bacteria culture medium, n=3.


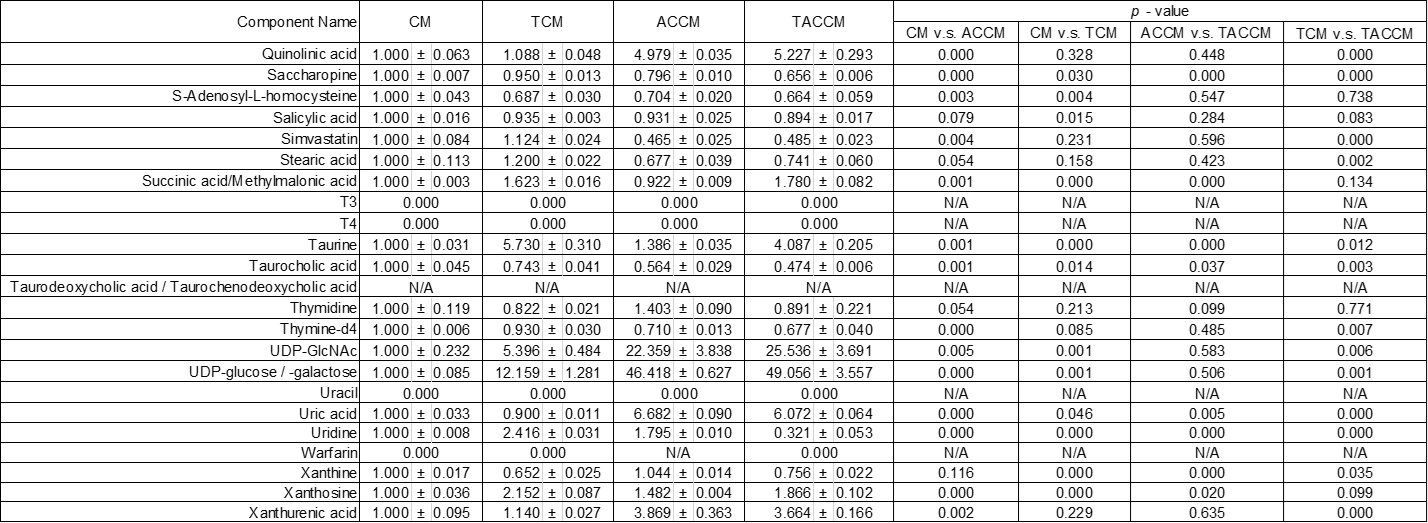


**Supplementary Table S3 (continued)**. Bioactive component analyses among 4 groups of supernatants by HILIC method in negative mode. CM: fresh bacterial culture medium; TCM: *Turicibacter* culture medium; ACCM: fresh medium with AC; TACCM: *Turicibacter* culture medium with AC. Data was presented as relative mean AUC ± SEM compared to fresh bacteria culture medium, n=3.
